# Vertical farming limitations and potential demonstrated by back-of-the-envelope calculations

**DOI:** 10.1093/plphys/kiaf056

**Published:** 2025-04-02

**Authors:** Samuel J Lovat, Εlad Noor, Ron Milo

**Affiliations:** Department of Plant and Environmental Sciences, Weizmann Institute of Science, Rehovot 7610001, Israel; Department of Plant and Environmental Sciences, Weizmann Institute of Science, Rehovot 7610001, Israel; Department of Plant and Environmental Sciences, Weizmann Institute of Science, Rehovot 7610001, Israel

## Abstract

Improving food security and reducing the environmental footprint of food production is urgently needed to satisfy the growing global population in a time of climate, biodiversity, and water pressures. Indoor vertical farming is largely independent of environmental conditions and is reported to reduce the land and water required for food production. However, vertical farming requires large amounts of energy. Based on the vertical farming energy cost, we derive from basic considerations a current minimum cost of ≈$10/kg dry plant matter. Vertical farming is therefore not currently competitive with dried cereals or pulses (e.g. wheat, rice, and soybeans). We also show limited current competitiveness for products like tomatoes and lettuce, despite a low dry matter content. Whereas the environmental implications of vertical farming depend on the electricity source. Using the average newly installed electricity mix in recent years (predominantly solar and wind, with some coal, natural gas, and bioenergy), vertical farming could substantially increase greenhouse gas emissions and has limited land benefits compared with conventional agriculture. Using exclusively electricity from photovoltaics, some environmental benefits could be achieved for crops with a low dry matter content like lettuce, but this is more limited for dried crops like wheat. The transparent calculations we provide here set out challenges for vertical farming and highlight that improvements in both the overall vertical farming energetic efficiency (≈1% to 2%), as well as low-impact electricity sources are needed in the future.

Advances Box

- Interest and investments in vertical farming have expanded as a possible approach to reliably produce food year-round and help reduce the environmental burdens of food production
- There are a growing number of questions about the economic viability of vertical farming and how this can be improved in the future
- Greater attention is also being given to the environmental footprint of vertical farming and whether it provides benefits over current modes of food production under different scenarios

## The promise and challenge of vertical farming

Climate change and future socioeconomic developments pose large risks to regional food security (Hultgren et al. 2022). Concomitantly, crop production uses one-tenth of global land area and dominates global water consumption (Hoekstra and Mekonnen 2012; Poore and Nemecek 2018). This is driving major biodiversity pressures and regional water shortages, leading to planetary instability (Richardson et al. 2023). Interest in vertical farming has been growing in recent years as a potential way to help meet these challenges, with several billion USD in funding in North America since 2015 alone (Western Growers and Berger 2022).

Vertical farming is a closed indoor production method in which plants are grown in vertically stacked layers. Typically, vertical farming relies solely on artificial light for plant growth and makes use of hydroponic systems with water recycling to improve water efficiency. Vertical farms therefore provide a precisely controlled growth environment independent of external environmental factors, enabling local food production year-round and improved food security. Vertical farming is also reported to require much less land and water per unit of product than conventional agriculture (Asseng et al. 2020; WayBeyond and Agritecture 2021; Western Growers and Berger 2022). However, substantial amounts of energy and other infrastructure are required, which have economic and sustainability ramifications that need to be accounted for.

The economic and environmental potential of vertical farming has been explored using sophisticated techno-economic and life cycle assessments (Eaves and Eaves 2018; Asseng et al. 2020; Blom et al. 2022; Martin et al. 2023). However, such assessments are limited in number, covering only specific regions and products. Exploring new scenarios (e.g. producing different crops, or future systems) in such detail requires time and information that is often not readily available. Here, we use back-of-the-envelope calculations (otherwise known as Fermi calculations) to quantify the economic and environmental implications of vertical farms using exclusively artificial light for plant growth across various scenarios. We compare these results with conventional open-field agriculture, which dominates global food production and its environmental impacts and is therefore the most relevant benchmark for assessing the large-scale environmental implications of vertical farming.

The implications of shorter supply chains through vertical farming are addressed in the conclusion. While comparisons with other innovations for improving global food production are of significant interest, such as greenhouses (or genetically engineered crops), they are beyond the scope of this analysis, which focuses on whether vertical farming could even replace conventional agriculture—the predominant mode of food production.

Our calculations provide a transparent analysis that is simpler to communicate and extract lessons from. This helps to assess the potential of vertical farming, including its current economic and environmental limitations and longer-term possible gains from technology maturation, which can be useful to explore already in the early stages of this technology.

### Vertical farms convert purchased electricity energy into food energy with an efficiency of ≈1% to 2%

Current vertical farms focus largely on leafy vegetables (WayBeyond and Agritecture 2021; Western Growers and Berger 2022). Yet, staple crops like wheat and rice dominate global cropland area and water usage (Mekonnen and Hoekstra 2011; Ritchie and Rosado 2023). Expanding vertical farming to staple crops may be an opportunity to reduce global environmental pressures from food systems. To explore the potential of producing any given crop, we focus on the amount of energy needed for vertical farms. Vertical farming energy use can be readily estimated (as shown in the following paragraphs) and is currently a major driver of both costs and environmental impacts (Asseng et al. 2020; Martin et al. 2023). Even without considering other factors like infrastructure and labor, whose impacts are highly variable and challenging to quantify, the absolute minimum cost and environmental footprint of vertical farming can be estimated based on energy requirements. This thereby helps to identify initial constraints and opportunities for vertical farming.

Energy use can be estimated using the expected energetic conversion efficiency of vertical farming. Energetic conversion efficiency is defined here as the ratio of energy available for human consumption stored in plant matter (energy out) to the total amount of purchased energy used over the same period of time (energy in). To evaluate what the energetic efficiency of vertical farming is, we start by focusing on what we expect to be the major source of energy demand—the power for the artificial lighting needed for plant growth. What is the efficiency of converting light into plant matter? This is not easy to derive from basic principles but has been done by breaking down the process of photosynthesis and agricultural production, which gives a maximum value of around 10% (Zhu et al. 2008, 2010) (we explore this in more detail in the future energetic efficiency of vertical farming section below). Other factors, such as the efficiency of converting electricity into light energy, temperature and humidity control, and other operational demands, also dictate the energetic needs of vertical farming.

To arrive at a rule of thumb for the overall electrical energy to edible plant matter conversion efficiency of vertical farming, we compiled values for the amount of purchased energy used in vertical farms from a non-exhaustive literature survey. We identified 6 life cycle assessment studies of operational vertical farms published since 2022 that report electricity use across lighting, climate control, and water pumping (see Supplementary Data Set 1). We subsequently converted electricity use data into the energetic efficiency of vertical farming by comparing the estimated energy in the final edible product (energy out) with the amount of electrical energy supplied (energy in) (see Supplementary Data Set 1; Supplementary Fig. S1). For example, Martin et al. 2023 report ≈5 million kWh of electricity usage in a commercial vertical farm producing ≈500,000 kg of lettuce annually, equaling 10 kWh of electricity per kg of lettuce. Lettuce has an energetic content of ≈120 kcal/kg (FAO 2001), which translates to ≈0.1 kWh chemical energy/kg lettuce (using 1 kcal = 4.2 kJ and 3,600 kJ = 1 kWh, or ≈1 kWh/1,000 kcal). Therefore, for every 100 units of electrical energy supplied in the analysis of Martin et al. (2023), approximately 1 unit is stored as edible dry plant matter. Across these recent life cycle assessments for operational vertical farms, the median and mean energetic conversion efficiency of vertical farms is ≈1%.

Expanding the number of studies considered (n = 19) to include modeling work and small-scale test sites across a range of regions and crops, the median and mean energetic efficiency of vertical farming remains at ≈1%, with a range of 0.4% to 1.9% (Supplementary Fig. S1; Supplementary Data Set 1). Due to the order of magnitude approach, we did not adjust these studies to achieve consistent reporting of electricity use data for all production stages (e.g. climate control, water pumping, etc.), but all studies reported electricity use for lighting, which is the major source of electricity demand (Graamans et al. 2018; Weidner et al. 2021; Blom et al. 2022). Ultimately, we conclude that 1% to 2% is a current best practice rule of thumb for the total energy efficiency of vertical farming. Future developments discussed later might increase this by up to several-fold to ≈10%.

### Current electricity costs associated with vertical farming limit competitive production of staple crops and even vegetables

**Figure 1.**
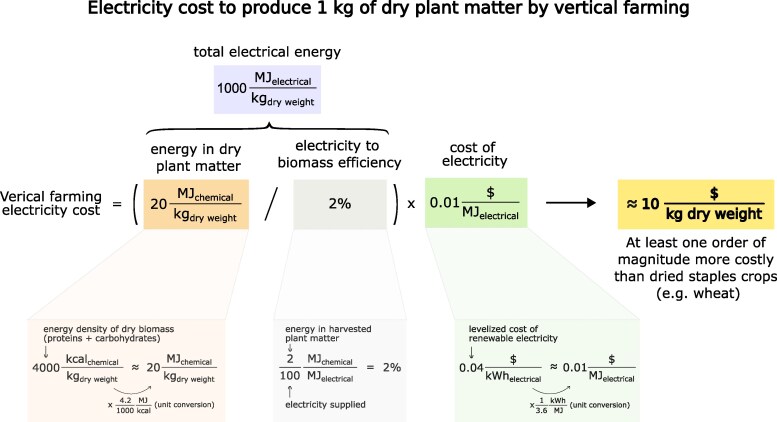
Summary of the cost of electricity for vertical farming to produce dry plant matter, which is analogous to dried staple crops like wheat and rice. The cost of electricity represents an absolute lower bound cost of production for vertical farming.

The energetic conversion efficiency factor is used to calculate the electricity use and minimum production cost of vertically farmed plant matter (summarized in Fig. 1). Given that vertical farming is in early development stages, we optimistically use the upper energetic efficiency value of 2% to allow for expected incremental improvements in the coming years. Note, the energetic efficiency is not dependent on the water content of different crops but changes with other crop-specific factors. For example, the fraction of plant matter grown that is edible (harvest index) varies between plant species. The harvest index of lettuce is close to 1, while for wheat it is close to one-half (Kobayashi et al. 2022). Thus, assuming all other factors are equal and dry matter is partitioned equally between harvested and unharvested plant fractions, the energetic efficiency of producing wheat would be roughly one-half that of lettuce. Because we assess here a best-case current scenario using an order of magnitude approach, we do not account for different harvest index values between crops, but this should be included in future detailed, crop-specific studies. More speculative, long-term transformative improvements in the vertical farming energetic efficiency are discussed below (in the future energetic efficiency of vertical farming section).

We start by considering the production of dry plant matter, which is largely composed of proteins and carbohydrates (De Vries et al. 1974) that have energetic contents of ≈4,000 kcal/kg (FAO 2003). If 2% of input energy is converted to edible plant matter, 200,000 kcal of purchased energy is needed per kilogram of dry plant matter. Using common unit transformations (1 kcal = 4.2 kJ and 3,600 kJ = 1 kWh, or ≈1 kWh/1,000 kcal), this corresponds to ≈250 kWh/kg of dry plant matter. As current farming directly harnesses sunlight, artificial lighting in vertical farming would increase electricity demands beyond present levels. We estimate the cost of this additional purchased electricity to be at least 4 cents per kWh, based on the recent break-even cost of producing 1 additional unit of electricity (so called, the levelized cost of electricity) (Luderer et al. 2022; Way et al. 2022; Bilicic and Scroggins 2023; Nijsse et al. 2023). Using this value, we arrive at an electricity cost of ≈$10/kg of dry plant matter grown using vertical farming. This value is before all processing and supply chain costs, which typically increase retail prices several-fold for foods produced by conventional agriculture (Yi et al. 2021).

Dried cereals and pulses, such as wheat, rice, and soybeans, are of major global importance. Farm-gate or producer prices for such crops are generally less than $1/kg worldwide and can be as little as $0.1/kg (FAO 2024a). This is 1 to 2 orders of magnitude less than the electricity cost for producing dried plant matter by vertical farming. Even disregarding all other costs (infrastructure, labor, etc.), this simple calculation shows that vertically farmed dried staple crops are unlikely to be economically viable in coming years, as long as current food prices, electricity costs, and vertical farming conversion efficiencies do not jointly change by 1 to 2 orders of magnitude.

One can also consider higher value products such as tomatoes or leafy vegetables like lettuce, which are largely water and have a dry matter content of only ≈5% (Popkin et al. 2010; Hou et al. 2020). Assuming plant energy content changes in proportion to dry matter content, vertical farming energy requirements also change proportionately with plant dry matter content. The back-of-the-envelope vertical farming energy cost for such products is therefore ≈5% of the cost derived for dry plant matter, or ≈$0.5/kg fresh leafy vegetables or tomatoes. This is in line with current producer prices for fresh tomatoes or lettuce of ≈$0.5/kg (FAO 2024a). However, we do not account here for vertical farming infrastructure, labor, ancillary energy requirements (e.g. office spacing, operations, refrigeration, etc.), and other costs. This makes vertically farmed tomato and leafy vegetable production also more expensive than conventional production. The price gap though is substantially smaller than for dried staple crops. With enough future developments, vertically farmed tomatoes and leafy vegetables could become price competitive with current in-field methods.

It is clear that the high electricity costs alone limit the current economic viability of large-scale food production by vertical farming, but certain factors could help change this. Vertical farming can capitalize on periods when electricity prices are below their daily average to power lighting for plant growth, although the benefits of this may be on the order of a few tens of percent (Avgoustaki and Xydis 2021). One can also consider future scenarios where electricity prices are markedly lower. For example, the break-even cost of electricity is projected to decline by up to several-fold in coming decades, reaching as little as $0.01 to $0.02/kWh in 2050 (Luderer et al. 2022; Way et al. 2022; Nijsse et al. 2023). This reduces the lower bound electricity costs for vertical farming to ≈$2 to $3/kg dry plant matter, or ≈$0.1 to $0.2/kg fresh tomatoes or leafy vegetables. Even at such low prices, vertically farmed dried staple crops are of limited viability, but there may be potential for higher value crops like leafy vegetables and tomatoes, although a more detailed assessment is needed. Additionally, vertical farming could be more competitive in regions with poor outdoor growing conditions and cheap, abundant electricity; or in years with poor weather conditions for plant growth, which may occur more frequently in future due to climate change (Hultgren et al. 2022). Although such considerations could be important for vertical farming, they are not considered in greater detail here as available information is limited.

### The environmental footprint of vertical farming depends on the electricity mix

**Figure 2.**
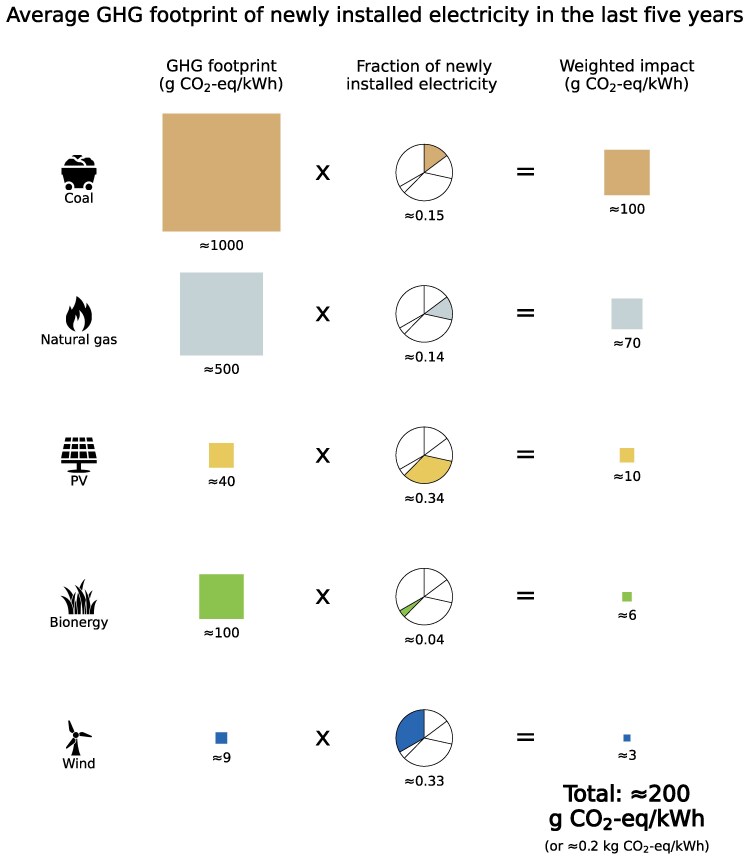
Average GHG footprint of newly installed electricity technologies in the last 5 years (2018 to 2023). This reflects the potential impact of electricity use in an average vertical farming system deployed in coming years. Combining the relative share of new electricity generation for each technology (Supplementary Data Set 2) with their literature-derived GHG footprint (Supplementary Data Set 3) gives the weighted average GHG footprint of 1 additional unit of electricity generated. Note that numbers have been rounded for ease, so calculations may not align exactly. Technologies such as nuclear, hydropower, and other renewables represent 1% or less of newly installed additions and have small environmental footprints; they therefore have a minor contribution to the weighted environmental footprint and have been excluded here for simplicity.

The environmental impact of food production is also of great importance. A lower bound estimate of the environmental impacts of vertical farming can also be derived from the electricity used. This of course is very sensitive to the electricity mix considered as the environmental footprint of different technologies (e.g. greenhouse gas footprint of fossil fuels compared with renewables) can vary by over an order of magnitude (Fig. 2; Supplementary Fig. S2). Vertical farms could use exclusively electricity from low-impact sources, such as photovoltaics (PVs) or wind turbines. However, the availability of these technologies and their rate of expansion are currently limited in many regions. This results in competition between sectors vying for low-impact electricity sources, including novel food technologies. Using low-impact electricity for vertical farms could therefore prevent other existing sectors from replacing high-impact electricity (e.g. fossil fuels), leading to a so-called environmental opportunity cost. However, increasing demand for current low-impact electricity sources could drive additional expansion of such low-impact technologies in future.

Given these complexities, we aim to quantify the environmental impacts of vertical farming using 2 different characteristic electricity mixes: first, a scenario based on the newly installed electricity mix in recent years; second, using just PVs.

Vertical farming would expand electricity demand beyond current levels. To reflect the electricity sources that may be installed to meet this additional demand, we use a mix based on technologies added in recent years to expand global electricity production. Because electricity mixes are rapidly diversifying (Ritchie and Rosado 2020), we consider newly installed electricity in the last 5 years (between 2018 and 2023 globally). This scenario is relevant for the average vertical farming systems deployed in coming years.

The second scenario, using electricity exclusively from PVs, could represent a current best-case scenario for vertical farming, considering the few regions where low-impact technologies are already abundantly available, such as Luxembourg or Denmark, which have a large share of electricity from PVs and wind turbines (Ritchie and Rosado 2020). Additionally, a PV scenario could inform the potential of vertical farming in the coming decades if electricity from PVs (in parallel to wind turbines) comes to dominate global electricity production (Luderer et al. 2022; Nijsse et al. 2023).

We emphasize that these scenarios are helpful for considering large-scale or nonspecific cases but represent 2 of many possible electricity mixes. For regions or scenarios where electricity mixes deviate substantially from those explored here, the electricity footprints used in the following calculations must be adjusted to account for this.

### Using the newly installed mix of electricity in vertical farming could increase environmental burdens compared with conventional farming

In the last 5 years, wind and solar accounted for one-third of new electricity production each (Supplementary Data Set 2). New electricity production from coal and natural gas further represented around 15% each, while bioenergy represented around 4%. Other technologies, such as nuclear and hydropower, had a share of 1% or less and have small environmental footprints (Hertwich et al. 2015; Jin et al. 2019; Lovering et al. 2022; UNECE 2022); they were therefore excluded from this scenario.

**Figure 3.**
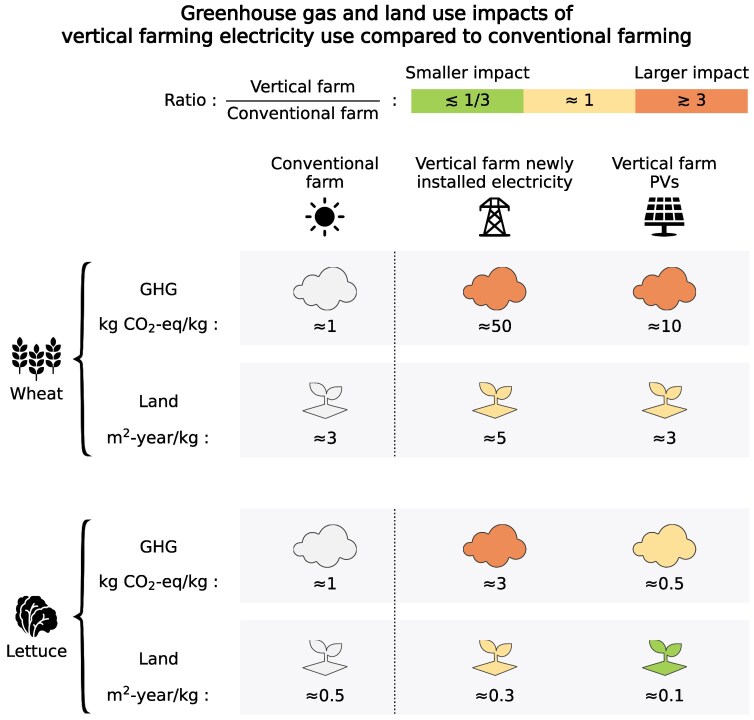
Summary of the GHG and land use impacts of conventional farms compared with electricity use of vertical farms alone. The median farm to retail impacts of conventional wheat and lettuce production were taken from Poore and Nemecek 2018 (see Supplementary Data Set 4 for further details). The color of the icons corresponds to the ratio of the environmental footprint of electricity use in vertical farming compared with conventional farming. Green shows at least a few-fold smaller relative environmental footprint from the electricity use of vertical farming, and orange shows a larger footprint. Absolute environmental impacts values of each scenario are shown below the icons. For the order of magnitude approach here, we used an energetic efficiency of 2% for all vertical farming scenarios. In reality, this value may vary for different crops due to differences in the fraction of edible plant matter, for example. If the energetic efficiency is rather 1%, for example, which is one-half the value used here, the environmental footprints would correspondingly double. Note, all numbers have been rounded for ease.

We estimated the weighted average footprint of 1 additional unit of electricity supply in the last 5 years as shown in Fig. 2 and Supplementary Fig. S2. This was done by combining the relative shares of newly installed technologies with the environmental footprint of each electricity source after excluding minor contributors (e.g. nuclear and hydropower). We considered 3 types of environmental impacts: greenhouse gas (GHG) emissions, land use, and water use. Since electricity impacts vary by location, we adopted characteristic regionally aggregated or global environmental electricity footprints based on data availability in the literature (Supplementary Data Set 3) to give a general scenario. The environmental implications of using this electricity mix for vertical farming are detailed below and are summarized in Fig. 3.

We estimated the weighted average GHG emissions footprint of new installations to the global electricity mix to be ≈200 gCO_2_-eq/kWh or ≈0.2 kgCO_2_-eq/kWh, which is largely driven by coal and natural gas, despite together representing only around one-third of this mix (Fig. 2). Combined with the electricity demand of ≈250 kWh/kg dry plant matter, this yields a GHG footprint of ≈50 kgCO_2_-eq/kg of dry plant matter (Fig. 3). This corresponds solely to emissions from vertical farming electricity supply. By comparison, GHG emissions of current dry staple crops, such as wheat and rice, are over an order of magnitude smaller, even when including emissions from land use change, transportation, retail, and supply chain food losses. For example, median farm to retail emissions from current wheat production are ≈1 kgCO_2_-eq/kg (Poore and Nemecek 2018). Considering instead vertically farmed tomatoes and leafy vegetables, which are largely water and hence require only ≈5% of the electricity needed to produce dry plant matter, GHG emissions would be on the order of at least 2 to 3 kgCO_2_-eq/kg. This is also higher than current median farm to retail emissions of ≈0.5 to 1 kgCO_2_-eq/kg for lettuce (Poore and Nemecek 2018) (Supplementary Data Set 4).

Electricity production also requires land, for example, for deploying solar panels and wind turbines (and of course mining coal, growing bioenergy crops, etc.). We estimated the weighted average land required for the newly installed electricity mix to be ≈0.02 m^2^-year/kWh, with bioenergy having a greatly outsized effect (Supplementary Fig. S2). The vertical farming land footprint for providing 250 kWh electricity/kg dry plant matter is therefore ≈5 m^2^-year/kg dry plant matter. This is similar to or a little larger than the land requirements for staple crops by conventional farming (e.g. ≈3 m^2^-year/kg wheat (Poore and Nemecek 2018)). For tomatoes and lettuce, the land footprint for electricity generation would be ≈5% of the value for dry plant matter production, at around 0.2 to 0.3 m^2^-year/kg. This is similar to, if not somewhat smaller than, the land currently needed (≈0.5 m^2^-year/kg) to produce lettuce (Poore and Nemecek 2018) (Supplementary Data Set 4), potentially offering small benefits, although a more detailed analysis is needed. Thus, although it is possible to grow plants much more densely in vertical farms, the electricity land footprint may negate this advantage, particularly for dried staple crops. However, compared with conventional agriculture, electricity generation does not require fertile land that is rich in biodiversity and stored carbon, and so the land use consequences of electricity use for vertical farming may be smaller.

Finally, the water used directly in vertical farming systems for plant growth and climate control is reported to be on the order of 10 L/kg of fresh lettuce (Carotti et al. 2023; Martin et al. 2023). This is several times smaller than the surface water and groundwater (collectively termed blue water) consumed on average by lettuce production in open fields (Mekonnen and Hoekstra 2011), mainly due to water recirculation and recovery in vertical farms. However, this does not include the blue water needed for electricity generation. For example, freshwater is used for cooling during fossil-fuel electricity generation, some of which is evaporated and lost from its original water basin and is no longer available for reuse (termed water consumption).

Vertical farming electricity use may increase blue water consumption compared with current dried staple crops, lettuce, and tomatoes when using the global average newly installed electricity mix (see Supplementary section S1). However, where water is used is much more important than how much is used, as the consequences of regional water use vary manifold depending on the location and its availability of water (Boulay et al. 2018). Electricity generation and vertical farming infrastructure are less location dependent than conventional agriculture. Moreover, electricity generation can be separated from vertical farms through long-distance electricity transmission. Vertical farming could therefore reduce regional water stress, especially if electricity generation is performed in water rich regions. Conversely, expanding electricity generation and vertical farming in regions with low water availability, such as the Middle East (Boulay et al. 2018), could increase regional water stress.

Overall, it seems that vertical farming using the global average mix of newly installed electricity additions may offer limited environmental benefits over current production methods, although reduced regional water stress may be possible.

### Vertical farming may offer some environmental benefits when using PV generated electricity

In the coming decades, electricity generation from PVs may increase greatly (in parallel to wind energy) (Luderer et al. 2022; Nijsse et al. 2023). PV electricity also mirrors conventional agriculture due to its reliance on sunlight as an energy source. We therefore assess the environmental implications of exclusively using electricity from PVs. The results are given below and summarized in Fig. 3.

The characteristic carbon footprint of electricity from PVs is currently ≈40 g CO_2_-eq/kWh or 0.04 kg CO_2_-eq/kWh (Bruckner et al. 2014; Asdrubali et al. 2015; Hertwich et al. 2015; NREL 2021). This is 5-fold lower than the footprint of the newly installed electricity mix used in the previous section (≈200 g CO_2_-eq/kWh; Fig. 2). Electricity use GHG emissions would therefore be ≈10 kgCO_2_-eq/kg dry plant matter (down from ≈50 kgCO_2_-eq/kg dry plant matter). This is still an order of magnitude larger than the current wheat farm to retail emissions. For lettuce and tomato production, however, the GHG footprint using PVs could decrease to ≈0.5 kgCO_2_-eq/kg. Although this does not include other aspects of vertical farming, this is similar to the ≈0.5 to 1 kgCO_2_-eq/kg currently emitted from the full life cycle of lettuce (Poore and Nemecek 2018) (Supplementary Data Set 4).

Utility-scale PV farms have a characteristic land footprint of ≈0.01 m^2^-year/kWh (Asdrubali et al. 2015; Hertwich et al. 2015; Lovering et al. 2022; UNECE 2022). It is possible to reach this value through a simple calculation, serving as a sanity check. Peak solar intensity is around 1,000 W/m^2^ (Blankenship et al. 2011). Considering changes in the angle of the sun, cloud cover, and day-night variations, global average solar intensity is ≈200 W/m^2^ (IPCC 2021). The amount of sunlight converted to electricity on solar farms is around 5% (Leger et al. 2021). Note, this is lower than the PV efficiency under standard laboratory conditions due to spacing between PV panels, imperfect solar tracking, and dust and debris covering solar panels in the field. Combining ≈200 W/m^2^ with an efficiency of 5% (1/20) and roughly 10,000 h in a year gives ≈100 kWh/m^2^-year or ≈0.01 m^2^-year/kWh. This is around one-half the value of the newly installed electricity mix as bioenergy is no longer included. The land footprint of vertically farmed dry plant matter using PVs would therefore be ≈2 to 3 m^2^-year/kg, which is similar to current wheat production (Poore and Nemecek 2018). The vertical farming land footprint for electricity use for tomatoes and lettuce would decrease to ≈0.1 m^2^-year/kg, which could offer benefits over current production, although a more detailed analysis is needed here.

Finally, the blue water footprint of PV electricity used for vertical farming would be smaller for wheat, lettuce, and tomatoes (see Supplementary section S1). However, as discussed in the previous section, the location of water use is much more important here than the amount of water used.

Overall, vertical farming using electricity from current PVs offers limited potential to reduce the environmental footprint of producing dry staple crops, such as wheat. For crops with a high-water content, land, water, and GHG emission benefits could be possible using electricity from PVs. However, a more complete life cycle assessment is needed here. Additionally, the intermittency of PV electricity must be addressed for this to be a realistic option. In this respect, energy storage, such as batteries, are rapidly scaling-up (IEA 2024a).

### The energetic efficiency of vertical farming could increase by up to several-fold in the future, reducing costs and environmental impacts

The expected energetic efficiency of vertical farming, as given above (2%), is a major limitation by driving high electricity use. Vertical farming is still in relatively early stages of maturity. Energetic efficiency could potentially increase several-fold in coming years. There are, however, biological limits to the efficiency of converting light energy into edible plant matter (Zhu et al. 2008, 2010), giving an upper-bound energetic efficiency for vertical farming.

**Figure 4.**
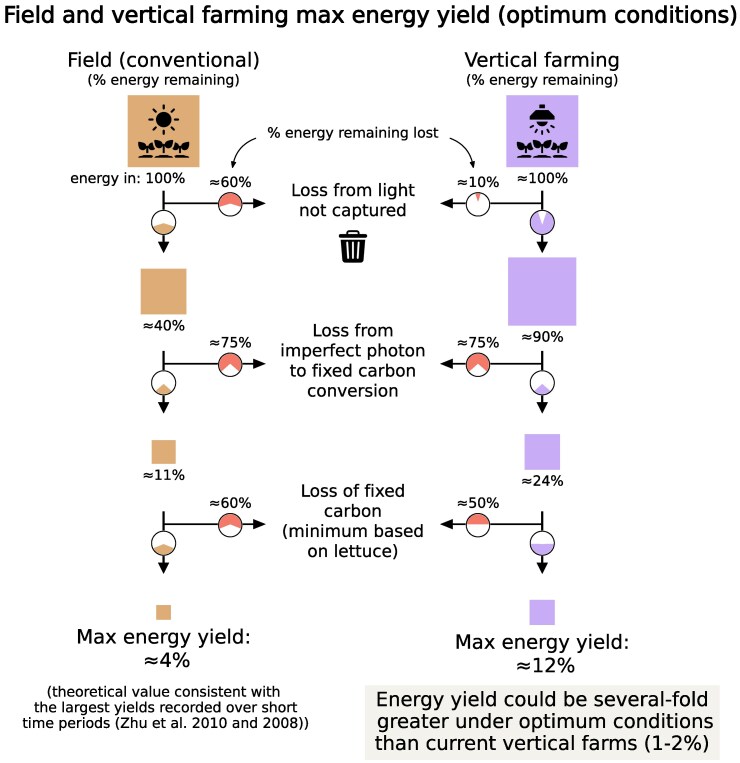
Summary of the maximum energy stored in edible plant matter under optimum conditions for C3 plants in conventional agriculture and vertical farms. Energetic losses (represented by the orange portions of the pie charts) are based solely on the biological limits for current plants, as analyzed in Zhu et al. (2008, 2010), ignoring energetic losses from other processes (e.g. converting electricity to light). Values given for energetic losses correspond to the percentage of energy remaining at a given stage that is lost. Energetic losses are combined into 3 sets of processes (light not captured, imperfect photon to fixed carbon conversion, loss of fixed carbon). Light not captured refers to non-photosynthetically active radiation, imperfect interception of light, and light reflected or transmitted. Imperfect photon to fixed carbon conversion includes relaxation of higher excited states of chlorophyll and losses associated with photon to carbohydrate conversion. Photorespiration wastes energy from photons but also releases CO_2_ and the potential to fix more carbon; it is therefore included under the loss of fixed carbon, together with respiration and inedible plant matter that is not harvested (otherwise known as the harvest index). For the energetic losses of the individual stages of edible plant matter production and further details, see Supplementary Fig. S3 and Supplementary Table S1. Energetic losses for C4 plants differ at some stages, but the maximum attainable yield is of a similar magnitude (see Supplementary Table S1).

The theoretical maximum energy yield for conventional agriculture has previously been explored in great detail (Zhu et al. 2008, 2010) and is a useful reference for assessing the biological limits in vertical farming. The maximum yield from full-spectrum sunlight in conventional agriculture for C3 plants is ≈4% due to energetic losses during photosynthesis and plant metabolism (Fig. 4, with a more detailed stepwise process and explanation given in Supplementary Fig. S3 and Supplementary Table S1). For example, at least two-thirds of energy is lost between the photosynthetic reaction centers and carbohydrate synthesis (Zhu et al. 2008).

The energetic losses in conventional agriculture help to appreciate that the maximum energetic yield in vertical farming under optimal conditions could be a few-fold larger (Fig. 4; Supplementary Fig. S3). For example, conventional agriculture relies on broad-spectrum sunlight, with one-half of all energy falling outside of wavelengths used in photosynthesis (Zhu et al. 2008). In vertical farming, only photosynthetically usable light needs to be generated, which could retain up to two-fold more energy, assuming zero energetic losses from conversion of electricity to light (Fig. 4; Supplementary Fig. S3; Supplementary Table S1). Additionally, elevated CO_2_ concentrations in vertical farming can suppress photorespiratory energy losses. This is discussed further in Supplementary Fig. S3 and Supplementary Table S1. Combining these changes raises the maximum achievable energetic efficiency to roughly 10% in vertical farming (Fig. 4; Supplementary Fig. S3). The exact value depends on the crop due to differences, for example, in harvest index, but we suggest this as a reasonable upper-bound yield across all crops.

The biologically achievable energetic efficiency of vertical farming is not fixed as plant engineering or optimizing conditions (e.g. CO_2_ concentration) could raise this ceiling. Recent advances in plant genome editing tools could be of benefit here (Chen et al. 2019). Additionally, because vertical farming is performed in a controlled environment, plant metabolism could be streamlined to increase efficiency by removing unnecessary features, such as mechanisms for dealing with light stress. However, this may take several decades due to difficulties of rewiring plant central metabolism (Zhu et al. 2010), combined with regulatory and social concerns about genetic engineering (Chen et al. 2019).

Other aspects of vertical farming, beyond biological limits, could also negatively impact the maximum energy yield. For example, converting electricity into light using light emitting diodes is not perfect and increases energy requirements by 2-fold in Graamans et al. (2018) due to a light emitting diode efficiency of ≈50%. Energy is also needed for other aspects of vertical farming, such as temperature regulation. Without considering these additional components, the roughly 10% energetic efficiency represents a simple and optimistic upper bound for vertical farming and is substantially larger than the current upper energetic efficiency of ≈2% given above.

An energetic efficiency of roughly 10% would lower the cost of supplying electricity to ≈$2/kg dry plant matter, which is still several-fold higher than current dried staple crops. However, for lettuce and tomatoes, the electricity cost would become ≈$0.1/kg, which could substantially increase the economic viability of producing such products by vertical farming. Further combining this with possible declines in electricity prices in the future means that vertical farming could become a more viable option for producing higher value crops with a low dry matter content in coming years.

Raising the energetic efficiency several-fold would also reduce the environmental impacts from electricity use by several-fold. This reduction is analogous to, if not larger than, the benefits seen in the previous section of switching from newly installed electricity to electricity generated by PVs. As with PV electricity, an energetic efficiency of roughly 10% could afford environmental benefits for producing tomatoes and leafy vegetables over conventional agriculture, but not dried staple crops. In the long term (several decades), energetic efficiency improvements together with low-impact electricity could further improve the environmental benefits from vertically farmed leafy vegetables and tomatoes. However, it is less clear whether such improvements would also lead to environmental benefits from producing dried staple crops by vertical farming. Ultimately, a more complete assessment of technological changes and non-electricity impacts is needed to better understand the potential of vertical farming in the long term compared with conventional agriculture.

## Conclusions

We derive here the minimum cost of vertical farming based on the cost of supplying electricity. For dried staple crops (e.g. wheat), we estimate that vertical farming will be over an order of magnitude more expensive than current production (≈$10/kg compared with less than ≈$1/kg), based on the expected upper-bound energetic efficiency of vertical farming in the the near future (≈2%). We also find that the absolute minimum cost of producing high-value vegetables with a low dry matter content, like lettuce and tomatoes, through vertical farming (≈$0.5/kg) is comparable with the cost of current conventional methods (also about $0.5/kg). Vertical farming therefore currently has limited economic competitiveness with conventional agriculture (see Outstanding Questions Box). In the coming decades, declining electricity costs and energetic efficiency improvements could greatly reduce vertical farming electricity use costs. Even still, large economic challenges would remain for the production of dried staple crops, but these advances could make vertical farming more economically feasible for products like tomatoes and lettuce.

Vertical farming could also be a viable competitor with greenhouse production (Eaves and Eaves 2018; Avgoustaki and Xydis 2020), especially as vertical farming technology matures. The global market value of greenhouse production is a few tens of billions of dollars annually (Mittal and Verma 2022; FMI 2024). This is 2 orders of magnitude smaller than the global value of conventional agriculture (FAO 2024b) and beyond the scope of our analysis. Still, this represents a substantial opportunity for individual vertical farming companies that could be explored further in a different context. Economic opportunities also extend beyond replacing food production to include production of pharmaceuticals and bioproducts from plants (Holtz et al. 2015) (see Outstanding Question Box).

When estimating the environmental footprint of vertical farms, the electricity mix considered is of central importance. This is both because of the high energy demand of vertical farming and limited availability of low-impact electricity, which creates an environmental opportunity cost between sectors competing for such sources. If the mix of newly installed electricity in recent years is used, vertical farming is predicted to substantially increase environmental pressures from food production, particularly the GHG emissions of producing dried staple crops, if this is pursued in the future. However, environmental benefits could be possible using either low-impact electricity or a higher energetic efficiency for crops with low dry matter content (e.g. lettuce and tomatoes). These contrasting effects reflect the sensitivity of vertical farming to the electricity supplied and show that especially in the longer term there is no clear rule of thumb for whether environmental benefits could be reached from vertical farming. Instead, more specific considerations are necessary, particularly given that electricity mixes could rapidly change in the coming decades as countries are diversifying energy sources. This nuance is also important for other technologies that heavily depend on electricity, such as electric vehicles (IEA 2024b). Ultimately, the framework provided here can help bring us toward a simple and transparent initial assessment of the environmental implications of different vertical farms.

Though we consider here constraints and levers for vertical farming, we are unable to account for some dimensions due to a lack of data for vertical farming systems and limited space. Vertical farms could be located close to the site of consumption and produce cleaner, higher quality crops with better nutritional properties (Managa et al. 2018). This could shorten supply chains and potentially lower their associated costs and increase product value. Since prices currently rise by several-fold beyond the farm-gate (Yi et al. 2021), the effect of shorter supply chains could be important, especially for higher-value crops where the price gap with conventionally produced products is smaller.

Much of the food produced globally is lost or wasted in current food supply chains or by consumers (OECD/FAO 2024; United Nations Environment Programme 2024). We did not include a detailed assessment of how this would change with vertical farming and potential shorter supply chains due to limited data availability. However, benefits would be relatively modest at best and would not significantly alter our outcomes. Higher quality and cleaner products, more flexible harvesting conditions and shorter supply chains could reduce food losses that arise from supply chains (e.g. processing, packing, transportation and distribution) and their associated environmental impacts. For conventionally produced foods, we included their supply chain environmental impacts up to and including the retail stage (Poore and Nemecek 2018). Whereas for vertical farming, we focused just on the environmental impacts of lighting for plant growth and were unable to include the supply chain impacts due to a lack of representative data. At best, including supply chain impacts for vertical farming would not change the environmental performance of vertical farms calculated in our analysis. More likely though, this would increase its relative footprint compared with conventional production.

Shorter supply chains could also provide fresher crops, which, combined with strategies such as high light intensity before harvest (Min et al. 2021), improves product shelf-life. At the consumer level (after supply chains), ≈20% of food purchased is wasted (United Nations Environment Programme 2024), contributing a similar proportion to total greenhouse gas emissions from the food sector (Zhu et al. 2023). Reduced consumer food waste could therefore contribute up to a 10% to 20% reduction in GHG emissions from the food sector under a very optimistic scenario. Such benefits though are relatively modest and on balance may be canceled out or reduced by the impacts of upstream supply chains from vertical farms.

Other aspects for future assessments of vertical farming include the flexible location of electricity generation and vertical farming, which could lower regional water, biodiversity, and climate pressures by avoiding environmentally important hotspots. Additionally, a mixture of artificial and natural sunlight could be considered for vertical farming, but this may not greatly reduce total energy use. This is because of the substantial lighting demand from plants grown in densely stacked layers, and energy increases for climate regulation due to less effective building insulation to allow for sunlight entry. Finally, the highly controlled indoor vertical farming environment could improve regional and urban food security and self-sufficiency while raising food safety standards and reducing exposure to pests and hence pesticide usage. Exploring these factors in the future (and additional questions raised in the Outstanding Questions Box) is important for a complete assessment of the benefits and constraints of vertical farming.

Using back-of-the-envelope calculations, we lay out in a transparent way some of the challenges and potential pathways for vertical farming, when more detailed assessments are limited. In the current situation, vertical farming is generally economically problematic, and environmental benefits may be possible only under limited circumstances. At the same time, future changes in energy systems, vertical farming efficiency, incentives, and food security could alter these results. The framework we offer here provides an approach to break down and explore such changes in a simplified manner and understand the key drivers of vertical farming and its limitations, helping to guide future efforts toward improved vertical farming and more sustainable food production.

Outstanding Questions Box

- How important will resilient and local food production be in the future, and could this lead to increased use of vertical farming?
- How much of a premium will consumers be willing to pay for fresher, higher quality crop products?
- How competitive is vertical farming in different scenarios compared with greenhouse production?
- How much can advances in optimization, plant genome editing, and other techniques improve the efficiency, cost, and environmental impact of vertical farming? Over what timescales will such innovations occur?
- What other areas of interest are there for vertical farming beyond food production? Is there a substantial opportunity and benefit from producing pharmaceuticals and other high-value products in such controlled environments?

## Supplementary Material

kiaf056_Supplementary_Data

## Data Availability

All of the data used here is given in the Supplementary data sets. The code used for the analysis has been deposited into GitLab (https://gitlab.com/milo-lab-public/vertical-farming.git).
