# Supplementary material for: Vertical farming limitations and potential demonstrated by back-of-the-envelope calculations
Source: Plant Physiol. 2025 Apr 2;198(3):kiaf056. doi: 10.1093/plphys/kiaf056 (PMC12225668; doi:10.1093/plphys/kiaf056)
Supplement: kiaf056_Supplementary_Data [file kiaf056_supplementary_data.zip › Supplementary_for_vertical_farming_limitations_and_potential_back_of_envelope_V3.pdf]

Supplementary Information for

Vertical farming limitations and potential demonstrated by back-of-the-envelope calculations

Samuel J Lovat[1], Elad Noor[1], Ron Milo[1*]

Affiliations:

[1] Department of Plant and Environmental Sciences, Weizmann Institute of Science, Rehovot, Israel, 7610001

**Corresponding author. Email:* ron.milo@weizmann.ac.il

This file includes:

Supporting text S1
Figures S1 to S3
Table S1
Supporting References

Other supporting materials for this manuscript include the following:

Datasets S1 to S4

Text S1. The water footprint of electricity use in vertical farming

Electricity use for vertical farming requires water. We outline here the water consumption footprint of vertical farming electricity use for the two scenarios explored in the main text (newly installed electricity mix and electricity exclusively from photovoltaics (PVs)). We emphasize though that the amount of water used can have highly variable consequences depending on the location and its availability of water.

The weighted average global blue water consumption (ground and surface water) for the newly installed electricity mix is ≈4 liters/kWh (see Dataset S3 and code). Given the requirement of ≈250 kWh/kg dry plant matter (see main text), the blue water footprint of electricity use for vertically farmed dry plant matter is therefore ≈1000 liters/kg. This is several-fold larger than the water consumed when producing dried staple crops (e.g., ≈300 liters/kg wheat (Mekonnen and Hoekstra, 2011)). For tomatoes and lettuce, the blue water consumption from electricity supply for vertical farming would be 20-fold smaller at ≈50 liters/kg. This is similar to the average blue water consumption for tomatoes (≈60 liters/kg) and lettuce (≈30 liters/kg fresh weight) (Mekonnen and Hoekstra, 2011).

Whereas the average blue water consumption of electricity from PVs is around ≈0.4 liters/kWh (Jin et al., 2019). This is an order of magnitude lower than the average new electricity additions mix (≈4 liters/kWh). Electricity use for vertical farming would therefore have a footprint of ≈100 liters/kg dry plant matter, which is less than current wheat production, for example (≈300 liters/kg wheat (Mekonnen and Hoekstra, 2011)). For vertically farmed fresh tomatoes and lettuce, the water footprint of electricity would be 5% of dry plant matter at ≈5 liters/kg. This is smaller than the average blue water consumption for tomatoes (≈60 liters/kg fresh weight) and lettuce (≈30 liters/kg fresh weight) (Mekonnen and Hoekstra, 2011).

Factors partially or not included in the water footprints above include the impact of using seawater (which is excluded from blue water use) instead of freshwater in power plants and the water consumed to supply the energy invested in crop production. However, these factors have at most a relatively modest effect and do not alter our analysis, as outlined in the following text.

Globally, around 70% of the water consumed in thermal power plants comes from freshwater sources, with the remaining 30% sourced from seawater (Lohrmann et al., 2019). Thermal power plants are used for generating electricity from coal, natural gas, oil, biomass and nuclear, which represent the majority of global electricity generation (Ritchie and Rosado, 2020). It is unclear how well the division between thermal power plants using freshwater and seawater is represented in the average blue water (which represents just freshwater and not seawater) footprint values used here. If values for thermal power plants consuming freshwater are overrepresented, then we would be overestimating the global average water footprint of electricity generation. However, this effect would reduce the water footprint values used here for vertical farming electricity use by up to a few tens of percent and does not alter the outcomes of our order of magnitude analysis.

With regards to the blue water consumed for supplying the energy used in agriculture, this is not accounted for in the water consumption values from Mekonnen and Hoekstra, 2011 adopted here. These values include just the water consumed in the field (e.g. for irrigation). However, the water footprint of energy use is negligible, as shown in the following calculation. Hoekstra and Mekonnen, 2012 estimate that on average ≈900 billion m^3 blue water/year was consumed globally for crop production in the years 1996-2005. By comparison, the industrial sector, which includes the total energy sector, consumed ≈40 billion m^3 blue water/year in the same period (Hoekstra and Mekonnen, 2012). Of the total primary energy generated globally, ≈10% is used for the entire food sector, including supply chains and household use (Usubiaga-Liaño et al., 2020). Only around one-third of food sector energy use is for producing plant-based products and on average ≈10% of food energy used is for the agricultural stage, with most used in other stages of food supply chains (Usubiaga-Liaño et al., 2020). Conservatively assuming that all industrial water use is for energy production, the water footprint associated with the energy used for production of plant products in agriculture is thus ≈0.1 billion m^3/yr (i.e. 40 billion m^3/yr x 10% x 30% x 10%). This is four orders of magnitude less than the water used for crop irrigation in the field and can therefore be ignored.

Supplementary figures

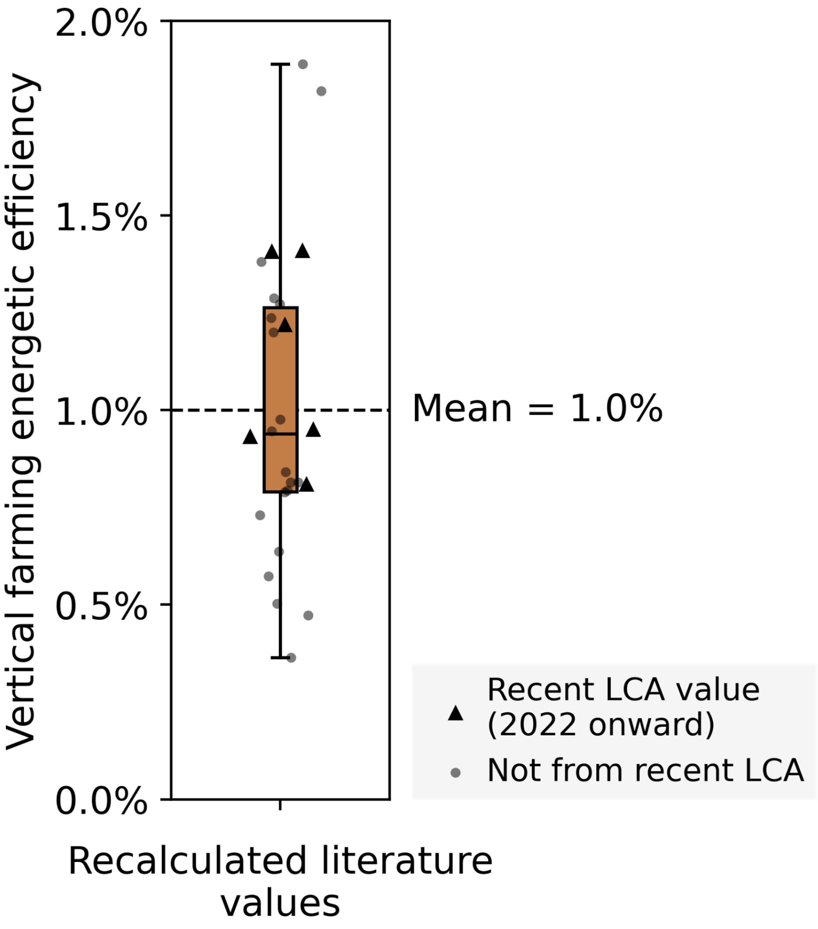

Figure S1. Energetic efficiencies of vertical farming systems reported in the literature. *Electricity requirements for commercial, small-scale and simulated vertical farms were collected from 19 studies, identified from a non-exhaustive literature survey, across different crops and regions (giving a total of 26 values). Electricity requirements (energy in) were converted into an energetic efficiency value by assuming the amount of chemical energy stored in the plant product (energy out). Values from recent life cycle assessment (LCA) studies from 2022 onwards are marked with triangles, corresponding to 6 out of the 19 studies identified. For further details, see Dataset S1. The box plot center line corresponds to the median; box limits are the upper and lower quartiles; whiskers refer to minimum and maximum values.*

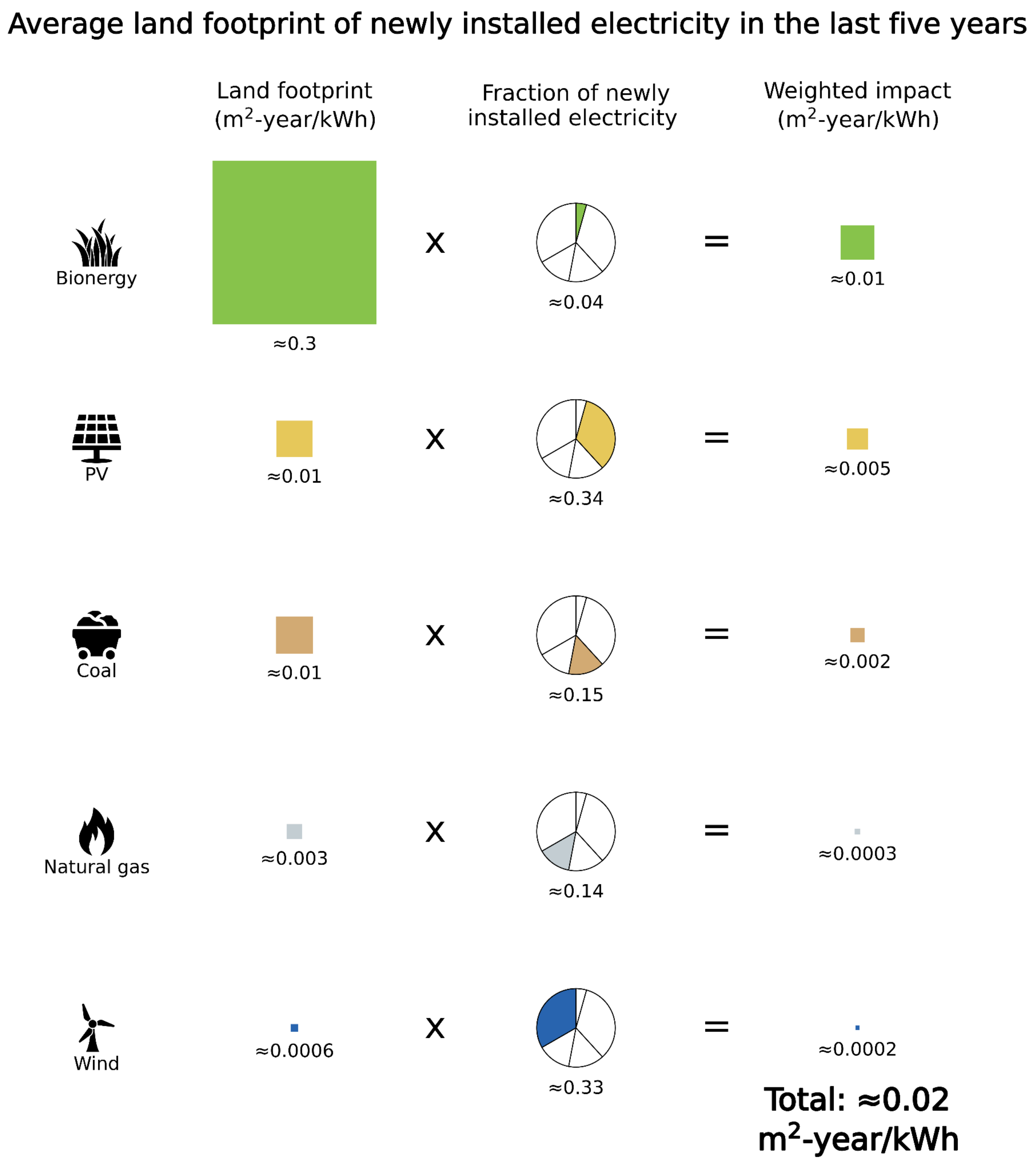

Figure S2. Average land use of the global newly installed electricity mix (2018-2023).
See caption of Fig. 2 for further details. If bioenergy is excluded, this total average land footprint of newly installed electricity would be ≈0.01 m²-year/kWh instead of ≈0.02 m²-year/kWh.

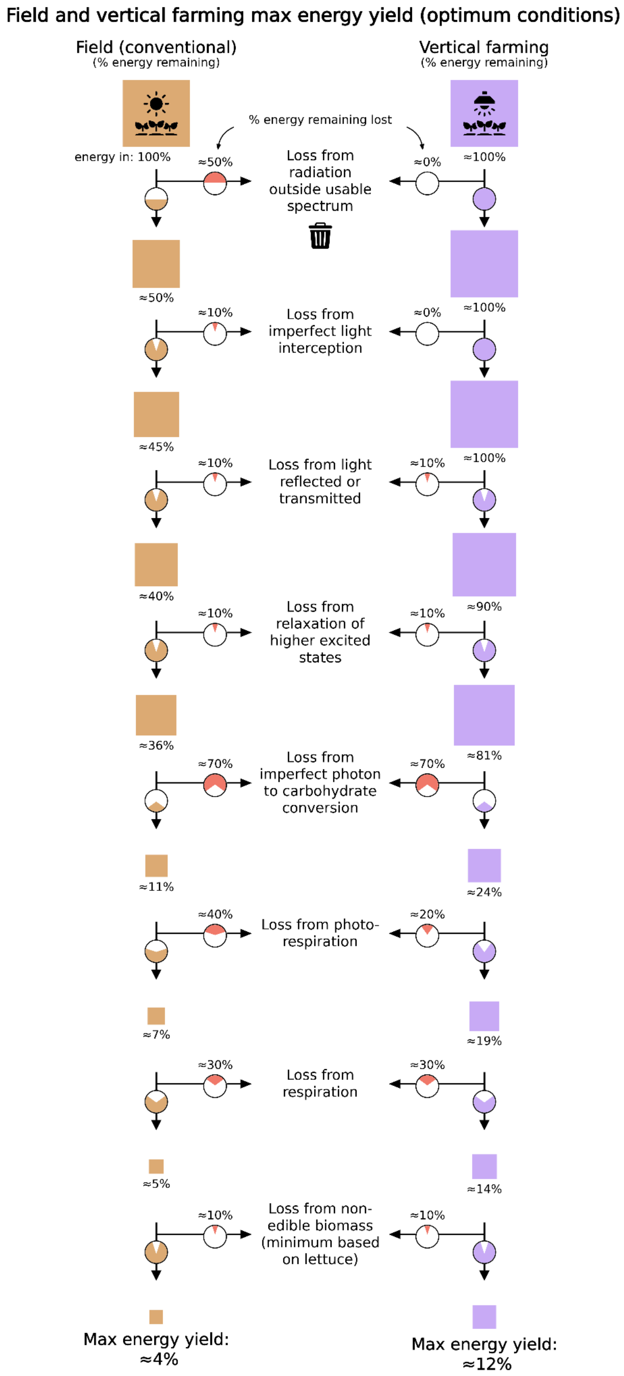

Figure S3. Maximum energy yields for conventional and vertical farms under optimum conditions with minimum energy losses. *Step-wise summary of the minimum energy losses for C3 plants in conventional and vertical farming assuming optimum conditions and their final maximum energy yields. Energetic losses are based on Zhu et al., 2008 and Zhu et al., 2010 and correspond to the percent of remaining energy lost at that given step. We consider here a theoretical optimum state constrained only by biological limits, thus assuming perfect conversion of electricity to artificial light and no energy needed beyond lighting. This gives an optimistic upper-bound value for the maximum energetic efficiency of vertical farming in the absence of major genetic engineering of plants. Detailed assumptions for each stage are given in Table S1.*

Table S1. Detailed assumptions of maximum energetic yields under optimum conditions with minimum energy losses for each stage of conventional (field) and vertical farming

Energy loss	Amount lost	Notes and assumptions
Radiation outside usable spectrum	Field: ≈50%	The energy source is sunlight, which contains a spectrum of wavelengths, many of which are outside the usable range for photosynthesis. The proportion of total incoming solar energy that is incapable of driving photosynthesis is around a half (Zhu et al., 2008).
	Vertical farm: 0%	With artificial lights (e.g. light emitting diodes or LEDs), it is possible to generate only photosynthetically active wavelengths. Given we are considering a theoretical maximum state in the future, constrained only by biological limits, we assume perfect efficiency of electricity to artificial lighting. We therefore assume no losses at this stage for vertical farming.
Imperfect light interception	Field: ≈10%	Not all photosynthetically active radiation is intercepted by plants as crops do not capture all radiation between sowing and plant canopy closure, leading to energy losses of at least ≈10% (Zhu et al., 2010).
	Vertical farm: 0%	Given the highly controlled conditions, we assume that light application can be optimized to change the light intensity and areas illuminated over time to minimize or eliminate unabsorbed light at all plant growth stages.
Light reflected or transmitted	Field: ≈10%	Of the light intercepted, not all is absorbed as some is reflected or transmitted. In conventional agriculture, this leads to a loss of at least ≈10% of the incoming energy (Zhu et al., 2010).
	Vertical farm: ≈10%	The photosynthetically active wavelengths used in vertical farming may be different to conventional agriculture. Indeed, a mixture of just red and blue light may be used (Pennisi et al., 2019), both of which are absorbed with an efficiency of ≈90% (Liu and van Iersel, 2021), resulting in similar losses to conventional agriculture.
Relaxation of higher excited states	Field: ≈10%	The average energy of photosynthetically active photons from sunlight is higher than the minimum energy needed to drive photosynthesis, resulting in relaxation of higher excited states of chlorophyll upon photon absorption, leading to energy losses of ≈10% to 20% (Zhu et al., 2008).
	Vertical farm: ≈10%	Similar to conventional agriculture, using higher energy blue (and red) photons in vertical farming leads to energy losses. We assumed that the ratio of red to blue light used in vertical farming will be around 3:1 (Carotti et al., 2024, 2023). This ratio is important for plant growth and producing high quality products (van Delden et al., 2021). Based on this, the energy of the average photon in vertical farming can be calculated using hc/λ, where h is Planck's constant (6.626×10^{-34} J s), c is the speed of light (3×10^{17} nm s^{-1}) and λ is the wavelength (in nm). Characteristic wavelengths of ≈450 nm and ≈650 nm are assumed for blue and red light respectively. This gives an average energy of $\approx 3.3 \times 10^{-19}$ J per photon or ≈200 kJ/mol of photons. This is similar to the average energy per mole of photosynthetically active photons from sunlight (Zhu et al., 2008), so we use the same value as conventional farming (≈10% to 20%).

Imperfect photon to carbohydrate conversion	Field: ≈70%	A minimum of eight photons in C3 plants are needed per mol of CO_2 fixed. Given the average energy of photosynthetically active photons from sunlight is ≈200 KJ/mol, a total of ≈1600 kJ of energy is needed per mol of CO_2 fixed. The energy stored per unit of carbon fixed is however only ≈500 kJ/mol (Zhu et al., 2008), resulting in a loss of ≈70% of energy in both current and vertical farming systems. For C4 plants of the NADP–Malic Enzyme subtype (not shown in the figure), almost 80% of the remaining energy is lost when converting photons to carbohydrates (Zhu et al., 2008).
	Vertical farm: ≈70%	Same as conventional agriculture.
Photorespiration	Field: ≈40%	Because of the imperfect selectivity of Rubisco, which catalyzes carbon fixation, additional energy is lost via photorespiration. The extent of photorespiration depends on the temperature and CO_2 concentrations present. Assuming a constant growing temperature of 25ºC in conventional farming and a CO_2 concentration of around 400 ppm, ≈40% of the remaining energy is lost (Zhu et al., 2008). For C4 plants (not shown here), the minimum energetic losses from photorespiration could be zero (Zhu et al., 2008).
	Vertical farm: ≈20%	In vertical farming, higher CO_2 concentrations can be used. Assuming a CO_2 concentration of 700 ppm, the energy lost is reduced to ≈20% (Zhu et al., 2008).
Respiration	Field: ≈30%	At least one-third of the remaining energy is lost due to respiration in (Zhu et al., 2008)
	Vertical farm: ≈30%	Assumed to be the same as conventional agriculture.
Non-edible biomass	Field: ≈10%	Finally, of the plant matter produced, not all is edible and can be harvested (the fraction of edible plant matter out of the total plant matter produced is termed the harvest index). The harvest index varies by crop, but to give the maximum possible yield across all crop species (or lowest energetic losses), we use the harvest index of lettuce, which is ≈90% (Kobayashi et al., 2022), resulting in a minimum loss of ≈10%.
	Vertical farm: ≈10%	Same as conventional agriculture.

References

Carotti, L., Pistillo, A., Zauli, I., Meneghello, D., Martin, M., Pennisi, G., Gianquinto, G., Orsini, F., 2023. Improving water use efficiency in vertical farming: Effects of growing systems, far-red radiation and planting density on lettuce cultivation. Agric. Water Manag. 285, 108365. https://doi.org/10.1016/j.agwat.2023.108365

Carotti, L., Pistillo, A., Zauli, I., Pennisi, G., Martin, M., Gianquinto, G., Orsini, F., 2024. Far-red radiation management for lettuce growth: Physiological and morphological features leading to energy optimization in vertical farming. Sci. Hortic. 334, 113264. https://doi.org/10.1016/j.scienta.2024.113264

Hoekstra, A.Y., Mekonnen, M.M., 2012. The water footprint of humanity. Proc. Natl. Acad. Sci. 109, 3232–3237. https://doi.org/10.1073/pnas.1109936109

Jin, Y., Behrens, P., Tukker, A., Scherer, L., 2019. Water use of electricity technologies: A global meta-analysis. Renew. Sustain. Energy Rev. 115, 109391. https://doi.org/10.1016/j.rser.2019.109391

Kobayashi, Y., Kotilainen, T., Carmona-García, G., Leip, A., Tuomisto, H.L., 2022. Vertical farming: A trade-off between land area need for crops and for renewable energy production. J. Clean. Prod. 379, 134507. https://doi.org/10.1016/j.jclepro.2022.134507

Liu, J., van Iersel, M.W., 2021. Photosynthetic Physiology of Blue, Green, and Red Light: Light Intensity Effects and Underlying Mechanisms. Front. Plant Sci. 12. https://doi.org/10.3389/fpls.2021.619987

Lohrmann, A., Farfan, J., Caldera, U., Lohrmann, C., Breyer, C., 2019. Global scenarios for significant water use reduction in thermal power plants based on cooling water demand estimation using satellite imagery. Nat. Energy 4, 1040–1048. https://doi.org/10.1038/s41560-019-0501-4

Mekonnen, M.M., Hoekstra, A.Y., 2011. The green, blue and grey water footprint of crops and derived crop products. Hydrol. Earth Syst. Sci. 15, 1577–1600. https://doi.org/10.5194/hess-15-1577-2011

Pennisi, G., Sanyé-Mengual, E., Orsini, F., Crepaldi, A., Nicola, S., Ochoa, J., Fernandez, J.A., Gianquinto, G., 2019. Modelling Environmental Burdens of Indoor-Grown Vegetables and Herbs as Affected by Red and Blue LED Lighting. Sustainability 11, 4063. https://doi.org/10.3390/su11154063

Ritchie, H., Rosado, P., 2020. Electricity Mix. Our World Data.

Usubiaga-Liaño, A., Behrens, P., Daioglou, V., 2020. Energy use in the global food system. J. Ind. Ecol. 24, 830–840. https://doi.org/10.1111/jiec.12982

van Delden, S.H., SharathKumar, M., Butturini, M., Graamans, L.J.A., Heuvelink, E., Kacira, M., Kaiser, E., Klamer, R.S., Klerkx, L., Kootstra, G., Loeber, A., Schouten, R.E., Stanghellini, C., van Ieperen, W., Verdonk, J.C., Vialet-Chabrand, S., Woltering, E.J., van de Zedde, R., Zhang, Y., Marcelis, L.F.M., 2021. Current status and future challenges in implementing and upscaling vertical farming systems. Nat. Food 2, 944–956. https://doi.org/10.1038/s43016-021-00402-w

Zhu, X.-G., Long, S.P., Ort, D.R., 2010. Improving Photosynthetic Efficiency for Greater Yield. Annu. Rev. Plant Biol. 61, 235–261. https://doi.org/10.1146/annurev-arplant-042809-112206

Zhu, X.-G., Long, S.P., Ort, D.R., 2008. What is the maximum efficiency with which photosynthesis can convert solar energy into biomass? Curr. Opin. Biotechnol., Food biotechnology / Plant biotechnology 19, 153–159. https://doi.org/10.1016/j.copbio.2008.02.004
